# Outcomes of Patients with Normal LDL-Cholesterol at Admission for Acute Coronary Syndromes: Lower Is Not Always Better

**DOI:** 10.3390/jcdd11040120

**Published:** 2024-04-15

**Authors:** Ivana Jurin, Anđela Jurišić, Igor Rudež, Ena Kurtić, Ivan Skorić, Tomislav Čikara, Tomislav Šipić, Diana Rudan, Šime Manola, Irzal Hadžibegović

**Affiliations:** 1 Department for Cardiovascular Diseases, Dubrava University Hospital, 10000 Zagreb, Croatia; ivanajurin1912@gmail.com (I.J.); andjelajurisic1@gmail.com (A.J.); tomislavsipic2@gmail.com (T.Š.); drudan@kbd.hr (D.R.); sime.manola@icloud.com (Š.M.); 2 Department of Cardiac and Transplant Surgery, Dubrava University Hospital, 10000 Zagreb, Croatia; rudi@kbd.hr; 3 Division of Cardiology, Department of Medicine, University Hospital Merkur, 10000 Zagreb, Croatia; ena.kurtic88@gmail.com; 4 School of Medicine, University of Zagreb, 10000 Zagreb, Croatia; ivanskoric123@gmail.com; 5 University North, Trg dr. Zarka Dolinara 1, 48000 Koprivnica, Croatia; 6 Faculty of Dental Medicine and Health Care, Josip Juraj Strossmayer University, 31000 Osijek, Croatia

**Keywords:** acute coronary syndrome, low density lipoprotein cholesterol, coronary revascularization, survival

## Abstract

Background and aim: There are few prospective data on the prognostic value of normal admission low-density lipoprotein cholesterol (LDL-C) in statin-naïve patients with acute coronary syndromes (ACS) who are treated with a preemptive invasive strategy. We aimed to analyze the proportion of patients with normal LDL-C at admission for ACS in our practice, and their characteristics and clinical outcomes in comparison to patients with high admission LDL-C. Patients and methods: Two institutions’ prospective registries of patients with confirmed ACS from Jan 2017 to Jan 2023 were used to identify 1579 statin-naïve patients with no history of prior coronary artery disease (CAD), and with available LDL-C admission results, relevant clinical and procedural data, and short- and long-term follow-up data. Normal LDL-C at admission was defined as lower than 2.6 mmol/L. All demographic, clinical, procedural, and follow-up data were compared between patients with normal LDL-C and patients with a high LDL-C level (≥2.6 mmol/L) at admission. Results: There were 242 (15%) patients with normal LDL-C at admission. In comparison to patients with high LDL-cholesterol at admission, they were significantly older (median 67 vs. 62 years) with worse renal function, had significantly more cases of diabetes mellitus (DM) (26% vs. 17%), peripheral artery disease (PAD) (14% vs. 9%), chronic obstructive pulmonary disease (COPD) (8% vs. 2%), and psychological disorders requiring medical attention (19% vs. 10%). There were no significant differences in clinical type of ACS. Complexity of CAD estimated by coronary angiography was similar between the two groups (median Syntax score 12 for both groups). There were no significant differences in rates of complete revascularization (67% vs. 72%). Patients with normal LDL-C had significantly lower left ventricular ejection fraction (LVEF) at discharge (median LVEF 52% vs. 55%). Patients with normal LDL-C at admission had both significantly higher in-hospital mortality (5% vs. 2%, RR 2.07, 95% CI 1.08–3.96) and overall mortality during a median follow-up of 43 months (27% vs. 14%, RR 1.86, 95% CI 1.45–2.37). After adjusting for age, renal function, presence of diabetes mellitus, PAD, COPD, psychological disorders, BMI, and LVEF at discharge in a multivariate Cox regression analysis, normal LDL-C at admission remained significantly and independently associated with higher long-term mortality during follow-up (RR 1.48, 95% CI 1.05–2.09). Conclusions: A spontaneously normal LDL-C level at admission for ACS in statin-naïve patients was not rare and it was an independent risk factor for both substantially higher in-hospital mortality and mortality during long-term follow-up. Patients with normal LDL-C and otherwise high total cardiovascular risk scores should be detected early and treated with optimal medical therapy. However, additional research is needed to reveal all the missing pieces in their survival puzzle after ACS—beyond coronary anatomy, PCI optimization, numerical LDL-C levels, and statin therapy.

## 1. Introduction

LDL-C is one of the most important modifiable risk factors for cardiovascular diseases (CVD) and the association between elevated levels of LDL-C and cardiovascular mortality has been known for decades [1,2]. Many studies have confirmed that the lower the LDL-C levels are, the lower the risk of complications of atherosclerosis, with no evidence of any clinically significant harm, no matter how low the LDL-C levels are [3,4]. Therefore, intensive LDL-C-lowering therapy with statins, ezetimibe, and proprotein convertase subtilisin-kexin type 9 (PCSK9) inhibitors became an established treatment option for patients with high-risk atherosclerotic cardiovascular disease [5,6,7].

However, some previous studies have demonstrated that lower LDL-C levels at admission in statin-naïve patients were associated with an increased risk of mortality following acute myocardial infarction (AMI) [8,9]. These observations led to a new term called “the lipid paradox” which refers to a paradoxical contradiction to the prevailing “cholesterol hypothesis” underlying the pathogenesis of atherosclerotic cardiovascular disease (ACVD) [10]. It has been postulated that this phenomenon might be related to malnutrition, cachexia, or generally worse clinical characteristics of patients [11,12], and inflammation [13]. A recent study in Japan [14] found that patients with low LDL-C levels at admission due to AMI had a significantly worse long-term prognosis than those with high LDL-C levels. Since ethnic differences between Asians and Caucasians have been reported in the outcomes after myocardial infarction [15,16] we sought to investigate the influence of admission LDL-cholesterol levels in statin-naïve patients on short- and long-term outcomes in ACS in our Caucasian cohort. In contrast to previous studies, our study was prospective in design, with all patients treated with invasive approach, and with known coronary artery disease complexity, treatment strategies, and outcomes.

We aimed to investigate the differences in characteristics, treatment strategies, and outcomes between the patients with low and high LDL-C at admission in order to test the independent relationship of low LDL-C at admission with mortality after ACS.

## 2. Patients and Methods

**Figure 1:**
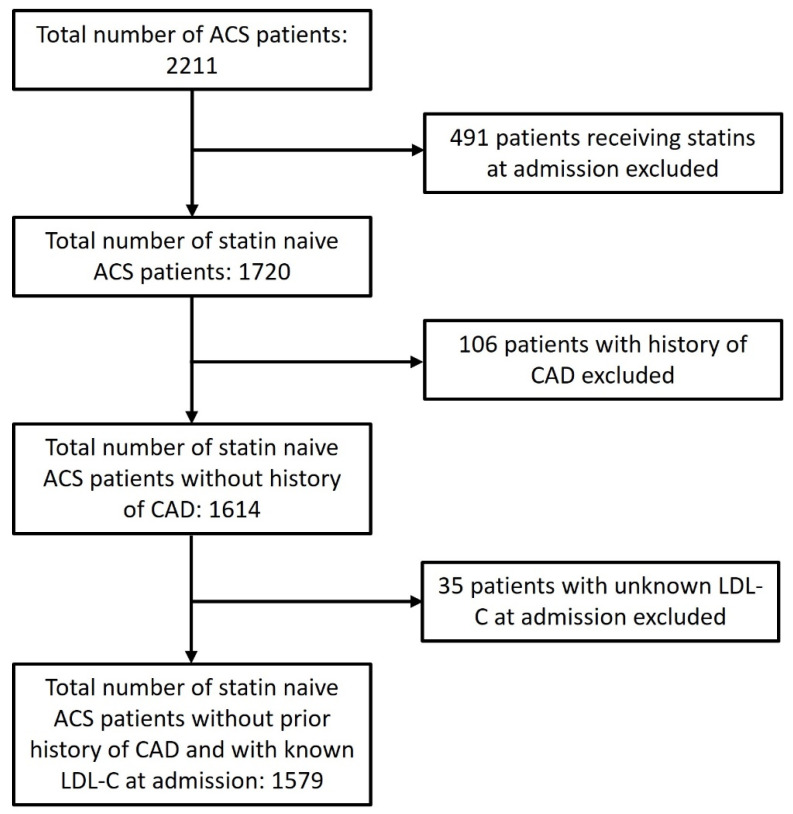
Patients’ selection flowchart.

Patients were collected from the all-comer ACS registry of two Croatian tertiary institutions, including patients with clear clinical, electrocardiographic, and laboratory signs of ST elevation myocardial infarction (STEMI) or non-ST elevation acute coronary syndrome (NSTE-ACS), as defined by the guidelines [17] who received coronary angiography, and were hospitalized between Jan 2017 and Jan 2023. Patient selection for this study is explained in detail in the flowchart (Figure 1). After excluding all patients with statin therapy, previously confirmed and treated CAD, or missing LDL-C levels at admission, there were 1579 patients left for analyses. All patients had relevant clinical and laboratory data noted on the day of hospitalization. LDL-C was determined in the clinical laboratory by subtracting the concentration of high-density lipoprotein cholesterol (HDL-C) and triglyceride level divided by five from the concentration of total cholesterol (Friedewald’s formula) [18]. If triglyceride levels were extremely high, where and when available, direct measurement of LDL-C was performed using standardized biochemical kits. If LDL-C levels were not available at admission, patients were excluded (Figure 1). Patients were divided into two groups regarding LDL-C at admission: the normal LDL-C group had LDL-C < 2.6 mmol/L, whereas the high LDL-C group had LDL-C ≥ 2.6 mmol/L. Renal function was expressed as creatinine clearance (CrCl) determined by the Cockroft–Gault equation [19]. Psychological disorders requiring medical attention were noted if the patient regularly took medication for a psychologic disorder or had a documented history of medical treatment by a psychiatrist. Their demographic and clinical data are displayed and compared in Table 1.

All participants had a coronary angiography performed during their hospitalization for ACS. Patients with suspected STEMI who did not have CAD confirmed by the urgent coronary angiography and were later diagnosed with Takotsubo cardiomyopathy, pericarditis, or any other non-CAD condition mimicking STEMI, were excluded from further analysis. All other patients with signs of myocardial infarction and no CAD confirmed using coronary angiography were included and diagnosed with myocardial infarction with no obstructive coronary artery disease (MINOCA). In case of culprit lesion confirmation, percutaneous coronary intervention (PCI) was performed. In the case of multivessel disease (MVD) the choice of ad hoc or elective total revascularization by PCI or coronary artery by-pass graft surgery (CABG) was determined by the operator or by the institutions’ heart team. Syntax score was used to objectively estimate the severity of coronary artery disease [20] and was calculated with the Syntax score official online calculator (https://syntaxscore.org/calculator/syntaxscore/frameset.htm (accessed on 14 March 2024)). All patients who survived until discharge received standardized treatment for ACS proposed by the guidelines [17] and had a detailed echocardiographic examination before discharge. Patients who died before discharge were also included in the analyses.

After discharge, patients were followed by routine clinic visits and by telephone calls. Telephone calls were used to determine relevant information and events that were not recorded during the routine clinic visits. Adherence to medical therapy was determined as low, moderate, or high according to patient-reported actual doses and regularity of statin and dual antiplatelet therapy after discharge. If patients reported irregular use and dose reduction in either of the two medications, adherence was considered low. Regular use with reduced dosing or irregular use of proposed doses of any of the proposed medication was defined as moderate adherence, whereas regular use of prescribed doses was defined as high adherence to medical therapy. The target LDL-C for patients treated from 2017 to 2019 was <1.8 mmol/L, whereas it was <1.4 mmol/L for patients treated after 2019 [21,22]. The main clinical endpoint for this study was overall survival. Routine clinical visits and telephone calls were used to determine causes of death. Cardiovascular death was defined if cause of death was identified as sudden death, myocardial infarction, heart failure, stroke, or pulmonary embolism. Other causes were considered to be non-cardiovascular deaths and were mostly malignancies, bleeding, or trauma. The study was conducted in accordance with the principles of the Declaration of Helsinki and the ethical standards of the committee responsible for patient data management.

## 3. Statistical Analysis

The normality of distribution of numerical variables were assessed with the Shapiro–Wilk test. Continuous numerical variables showed non-normal distribution and are presented as median and interquartile range, and the significance of differences between two groups was assessed with the Mann–Whitney test. Categorical variables are presented as frequencies and percentages, and the significance of differences between the groups was assessed with the χ2 test. Survival analyses were conducted with the Kaplan–Meier method. Survival curves were univariately compared using the Mantel–Cox log-rank test. Multivariate regression analyses were performed using the Cox regression. All variables that differed significantly (Table 1) were included in the multivariate Cox regression analyses. The final model was selected through a stepwise procedure with the “entry” and “stay” criterion of *p* ≤ 0.10. For LDL-C, two models were fitted, one with LDL-C as a categorical variable (<2.6 mmol/L or ≥2.6 mmol/L), and another with LDL-C as a continuous variable. The level of statistical significance was set at *p* < 0.05. Bonferroni correction for multiple simultaneous comparisons was used where appropriate. The analysis was performed with the IBM SPSS software, version 19 (IBM, Armonk, NY, USA).

## 4. Results

### 4.1. Overall Characteristics, Risk Factors, and Treatment Strategies

We analyzed the data of selected 1579 patients. The median age was 63 years (IQR 17 years) and the patients were predominantly male (1108 patients (70%)). There were 959 (61%) patients with STEMI, 549 (35%) patients with NSTE-ACS, and 71 (4%) patients with MINOCA. The median Syntax score was 12 (IQR 12.5), with 314 (20%) patients having MVD. Total revascularization by PCI or CABG within 6 months of initial presentation (either ad hoc or postponed) was achieved in 1125 (71%) patients.

There were 242 (15%) patients with LDL-C < 2.6 mmol/L (normal LDL-C) determined on the day of hospitalization. Patients in the normal LDL-C group differed significantly in some relevant demographic and clinical characteristics from patients in the high LDL-C group. They were significantly older, with lower creatine clearance levels (CrCl), and body mass index (BMI), and with significantly more cases of DM, PAB, COPD, and psychological disorders requiring medical attention (*p* < 0.05 for all comparisons; Table 1). There were no significant differences in other demographic and clinical characteristics, ACS type, vascular access, and CAD complexity measured by Syntax score. Regarding the timing of coronary angiography, patients with normal LDL-C were significantly less likely to receive coronary angiography within the first 24 h. Also, they had a significantly greater proportion of patients admitted with cardiogenic shock and/or cardiopulmonary resuscitation (Table 1).

The proportion of patients treated with PCI was significantly lower in the normal LDL-C group, with more cases treated with coronary artery by-pass grafting (CABG) or optimal medical therapy (OMT). However, there was no significant difference in the completeness of revascularization between the two groups. Patients with normal LDL-C had significantly lower LVEF at discharge (median LVEF 52%, IQR 17% vs. 55%, IQR 12%, *p* = 0.005, Mann–Whitney test). All patients with confirmed CAD after coronary angiography received dual antiplatelet therapy (DAPT) and statin therapy at discharge, except a minority of patients with contraindications or at physician discretion at discharge. The normal LDL-C group had significantly more patients with clopidogrel than ticagrelor or prasugrel prescribed at discharge. There were no differences in adherence to medical therapy between the two groups in the first year of follow-up. At 12 months after diagnosis of ACS, significantly more patients with normal LDL-C at admission had target LDL-C levels according to the guidelines available at the time of treatment (Table 2).

### 4.2. Clinical Outcome Associated with LDL-C at Admission

**Figure 2:**
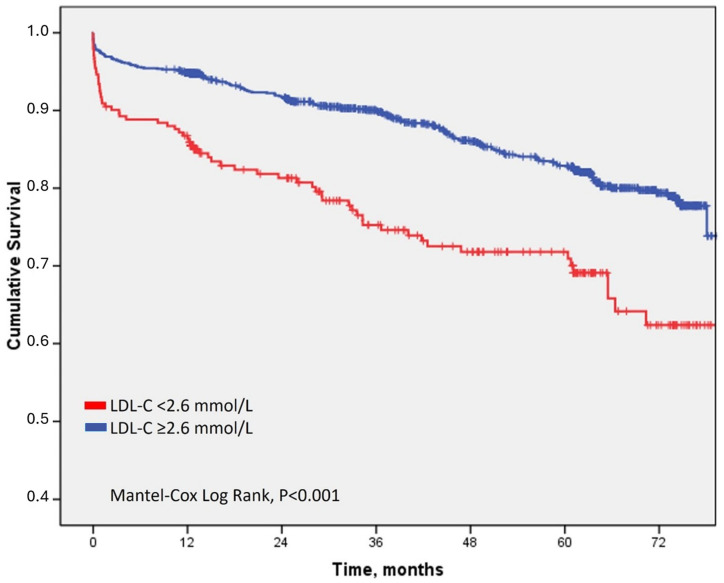
Overall survival after ACS regarding LDL-C at admission.

In-hospital mortality was higher in the normal LDL-C group (5% vs. 2%) with unadjusted relative risk for in-hospital death of 2.07, 95% CI 1.08–3.96 (*p* = 0.028). The median follow-up was 43 months. Clinical endpoint selected for assessment was death (overall survival). During the follow-up, 258 (16%) patients in the whole cohort died. When analyzing causes of death in the whole cohort, 173 (11%) of patients experienced a cardiovascular death (67% of all deaths during total follow-up). There were significantly more cardiovascular causes of death among the normal LDL-C group in comparison to the high LDL-C group (19% vs. 10%, respectively, Table 2). The patients in the normal LDL-C group experienced significantly shorter time-to-death than the patients in the high LDL-C group (Mantel–Cox Log rank, *p* < 0.001; Figure 2). Their unadjusted relative risk for death during follow-up was 1.86, with 95% CI 1.45–2.37 (*p* < 0.001). After adjusting for factors that differed significantly between the two groups (except cardiogenic shock and/or resuscitation, because it was biased for long-term survival, Table 1), an independent significant association with death during follow-up was confirmed for older age, higher BMI, lower EFLV at discharge, presence of psychologic disorders requiring medical attention, and LDL-C (both categorical: LDL-C < 2.6 mmol/L at admission associated with death during follow-up, and continuous: higher risk of death with lower LDL-C at admission) (Table 3).

## 5. Discussion

Our results showed that statin-naïve patients with normal LDL-C at admission for ACS have significantly worse prognosis in comparison to patients with high LDL-C, irrespective of their age, high-risk clinical characteristics and medical treatment, and revascularization strategies. To date, few studies have thoroughly investigated the correlation between baseline LDL-C concentration and long-term all-cause mortality in patients with acute coronary syndrome treated invasively. To the best of our knowledge, this study is the first to describe in detail the characteristics and invasive and medical treatment strategies in a relatively uniform cohort of patients with ACS and lower LDL-C at admission. We showed that patients with normal LDL-C at admission had more unfavorable clinical characteristics, although their burden of coronary artery disease calculated by Syntax score was not higher. Their higher in-hospital mortality was most probably due to more cardiogenic shock and CPR before coronary angiography. However, their long-term mortality was independently associated with low LDL-C levels at admission, even after correction for obvious clinical risk factors: age, diabetes mellitus, kidney function, and LVEF. Also, previous investigations tried to link the worse outcomes in patients with spontaneously low LDL-C with inflammation, or generally worse clinical characteristics leading to earlier non-cardiovascular death [11,12,13]. It must be stressed that patients in our relatively uniform ACS cohort experienced mostly cardiovascular deaths. Moreover, patients with normal LDL-C at admission had an even higher proportion of cardiovascular death in comparison to the high LDL-C group (45/63 (71%) vs. 128/194 (66%), respectively).

Earlier research [14] did not show statin therapy intensity at discharge, as well as adherence to lipid lowering therapy (LLT) and DAPT with the achievement of LDL-C target values during the long-term follow-up. A previous study comparing outcomes in patients with “spontaneous low” LDL-C at admission for AMI versus low LDL-C in patients already receiving statins at admission, also showed that patients with low “spontaneous” LDL-C had worse short-term outcomes [23]. However, that study reported that patients with “spontaneous” low LDL were more often discharged without statins, which was not the case in our study. Conversely, our study showed that patients with low LDL-C at admission did not differ in intensity of statin therapy at discharge and adherence during follow-up, and had significantly more patients achieving target LDL-C goals at 12 months. Nevertheless, their long-term mortality was significantly higher in comparison to the high LDL-C patient group.

What we also consider a novel finding in our study is a greater proportion of patients with psychological disorders requiring medical attention in the low LDL-C group. A recent study [24] found that higher LDL-C levels were associated with a lower risk of major depressive disorder (MDD), whereas one relatively recent case-control study found that higher LDL-C and total cholesterol levels and lower HDL-C levels were connected to anxiety disorder risk [25]. There are numerous gaps in our understanding of the role of cholesterol and its biosynthesis, so at this moment we could only suggest reasons for the described connection between “spontaneous” low LDL and psychological disorders in ACS patients [26]. Whatever the reason for that finding, it is clear from our data and from previous research [27], that psychological disorders are linked with worse outcomes after ACS. Our multivariate Cox regression showed that psychological disorders carried the greatest relative risk for shorter time-to-death after ACS (RR 1.81, 95% CI 1.27–2.57), irrespective of any other significant clinical feature. In addition to psychological disorders, patients in the normal LDL-C group were older and had more DM, which could also contribute to greater time to first medical contact for ACS, that could be linked with more cardiogenic shock and CPR before coronary angiography.

The present study has several limitations. First, this was an observational, prospective, dual-center registry study with a moderate sample size. Secondly, only admission LDL-C levels were obtained. It would be interesting to obtain previous data on LDL-C to investigate whether the patients with normal LDL-C experienced a recent drop in LDL-C levels or their LDL-C levels were normal for a longer period of adult life. That would identify patients with previous longer exposure to high LDL-C and a recent drop before an acute event, and probably help tailor their medical therapy in order to reduce future cardiovascular risk. We cannot speculate on the reasons why 15% of all ACS patients in this cohort had spontaneously normal LDL-C levels in the time of ACS presentation. However, this was dual-center real-life all-comer ACS registry, and no selection criteria were used other than previous statin therapy and on-admission LDL-C availability. Although there are studies with different thresholds of LDL-C used for similar analyses, LDL-C at 2.6 mmol/L was selected as a threshold for low vs. high LDL-C at admission in this study since it is mentioned in the guidelines as a threshold for primary prevention [22]. Third, we did not measure inflammation markers (i.e., hs-C reactive protein, interleukins, etc.) in a substantial number of patients in order to have enough reliable data for multivariate analyses. Acute and chronic inflammation has been associated with adverse outcomes after ACS, and that could be one of the missing links in the setting of normal LDL-C at admission for ACS [11,12,13,28,29]. Lastly, patients in the low LDL-C group had significantly more cases of DM, PAD, and lower CrCl, which could contribute to their residual risk beyond lipid control, although we proved an independent relationship in the regression analysis. These patients probably should have been put on high-intensity statin therapy before the first cardiovascular incident, irrespective of their seemingly acceptable LDL-C levels.

Additional research is needed to reveal all the missing pieces of the survival puzzle after ACS beyond coronary anatomy, PCI optimization, and numerical LDL-C levels achieved with statin therapy or other medication. In addition to inflammation, accumulating evidence from epidemiologic and genetic studies suggests that remnant lipoprotein (a) (Lp(a)), is causally related to residual risk in individuals already treated with statin therapy [30]. Dong et al. have shown that discordantly high Lp(a)/low LDL-C was associated with an unfavorable functional outcome, supporting the predictive potential of plasma Lp(a) after ischemic stroke, especially when discordant with LDL-C [31]. Determination of Lp(a) in plasma at the time of conducting this research was not yet incorporated into daily clinical practice. We can only assume, based on the growing body of evidence, that Lp(a) could be the missing piece in the puzzle of worse outcomes in these patients. Whether these patients with low “spontaneous” LDL-C would benefit from new therapies that are particularly successful in lowering Lp(a) remains to be confirmed in large randomized clinical trials. However, our study showed that global cardiovascular risk and screening for CAD should be evaluated more carefully in patients with other CVD risk factors and no overt dyslipidemia, in order to reduce the risk of acute or recurrent cardiovascular events.

## Figures and Tables

**Table 1:** Clinical characteristics of patients with ACS regarding LDL-C at admission.

Clinical Characteristic, Median (IQR) or Number (%)		LDL-C at Admission		*p* Value (Mann–Whitney or χ2 Test)
		Normal (<2.6 mmol/L) N = 242	High (≥2.6 mmol/L) N = 1337	
Age, years		66 (19)	62 (16)	<0.001
Male sex		172 (71)	936 (70)	0.761
Creatinine clearance, ml/min		71 (43)	81 (32)	<0.001
Arterial hypertension		909 (68)	180 (74)	0.057
Diabetes mellitus		64 (26)	226 (17)	0.001
LDL-C, mmol/L		2.1 (0.6)	3.5 (1.2)	<0.001
HDL-C, mmol/L		1.0 (0.3)	1.2 (0.3)	<0.001
NonHDL-C, mmol/L		2.8 (0.6)	4.6 (1.3)	<0.001
Triglycerids, mmol/L		1.3 (0.9)	1.6 (1.2)	<0.001
Peripheral artery disease		33 (14)	114 (9)	<0.001
Chronic obstructive pulmonary disease		19 (8)	29 (2)	<0.001
Current or previous smoking		112 (46)	663 (50)	0.454
Body mass index, kg/m^2^		27.7 (5.7)	28.6 (5.7)	<0.001
Psychological disorder		46 (19)	145 (10)	<0.001
ACS type	STEMI	139 (57)	820 (61)	0.536
	NSTE-ACS	92 (38)	460 (35)	
	MINOCA	11 (5)	57 (4)	
Timing of coronary angiography	≤24 h	175 (72)	1122 (84)	<0.001
	>24 h	67 (28)	215 (16)	
Wrist vascular access		194 (80)	1137 (85)	0.177
Left anterior descendent as infarct related artery		94 (39)	526 (39)	0.952
Syntax score		12 (13)	12 (12.5)	0.996
Mulltivessel disease		51 (21)	263 (20)	0.878
Cardiogenic shock and/or cardiopulmonary resuscitation		40 (17)	93 (7)	0.001

IQR—interquartile range, LDL-C—low density lipoprotein cholesterol, STEMI—ST elevation myocardial infarction, NSTE-ACS—non-ST elevation acute coronary syndrome, MINOCA—myocardial infarction with no obstructive coronary artery disease.

**Table 2:** Treatment strategies and outcomes during hospitalization and after discharge regarding LDL-C at admission.

Treatment and Outcome Variables, Number (%) or Median (IQR)		LDL-C at Admission		*p* Value (χ2 or Mann–Whitney Test)
		Normal (<2.6 mmol/L) N = 242	High (≥2.6 mmol/L) N = 1337	
Treatment strategy	PCI	192 (79)	1180 (88)	0.001
	CABG	18 (8)	59 (5)	
	OMT	32 (13)	98 (7)	
Complete revascularization		163 (67)	962 (72)	0.085
LVEF at discharge, %		52 (17)	55 (12)	0.005
DAPT at discharge	Ticagrelor	140 (58)	920 (69)	<0.001
	Prasugrel	14 (6)	154 (12)	
	Clopidogrel	72 (30)	218 (16)	
	No DAPT	16 (6)	45 (3)	
Statin at discharge	Maximal dose	223 (92)	1282 (96)	0.348
	Submaximal dose	13 (5)	45 (3)	
	No statin	6 (3)	10 (1)	
Adherence to medical therapy after discharge	Low	48 (24)	247 (20)	0.475
	Moderate	66 (33)	397 (32)	
	High	85 (43)	588 (48)	
LDL-C target goal achieved at 12 months		88 (36)	339 (25)	<0.001
Death, in-hospital		12 (5)	32 (2)	0.083
Death, overall		63 (26)	194 (15)	<0.001
Death, causes	Cardiovascular	45 (19)	128 (10)	<0.001
	Other	15 (6)	58 (4)	
	Unknown	3 (1)	8 (1)	

IQR—interquartile range, LDL-C—low density lipoprotein cholesterol, PCI—percutaneous coronary intervention, CABG—coronary artery by-pass graft, OMT—optimal medical treatment, LVEF—left ventricular ejection fraction, DAPT—dual antiplatetet therapy.

**Table 3:** Cox proportional hazard regression analysis of the impact of relevant clinical characteristics according to univariate analysis and LDL-C at admission on death during follow-up.

Variable	Multivariate Cox Regression, Death, HR (95% CI)
Age, continuous	1.056 (1.042–1.070) *
Creatinine clearance, continuous	1.002 (1.000–1.004)
Diabetes mellitus, categorical	0.997 (0.707–1.406)
Peripheral artery disease, categorical	1.273 (0.862–1.879)
Chronic obstructive pulmonary disease, categorical	0.888 (0.478–1.895)
Body max index, continuous	1.035 (1.002–1.069) *
Psychological disorder, categorical	1.810 (1.271–2.577) *
LVEF at discharge, continuous	0.940 (0.928–0.953) *
LDL-C, categorical (<2.6 mmol/L at admission)	1.483 (1.052–2.091) *
LDL-C, continuous	0.819 (0.710–0.944) *

LDL-C—low density lipoprotein cholesterol, HR—hazard ratio, CI—confidence interval, LVEF—left ventricular ejection fraction. * Statistically significant impact, *p* < 0.05.

## Data Availability

The data presented in this study are available on request from the corresponding author due to legal restrictions.
